# Functional activity of anti-endothelin type A receptor antibodies as a predictive tool of allograft injury in pediatric kidney transplantation

**DOI:** 10.3389/fimmu.2026.1925567

**Published:** 2026-10-22

**Authors:** Marta Vadori, Ezgi Demirkilic, Corrado Lanera, Benedetta Antoniello, Susanna Negrisolo, Emanuele Vianello, Caterina Di Bella, Elisa Benetti, Emanuele Cozzi

**Affiliations:** 1 Department of Cardiac, Thoracic, Vascular Sciences and Public Health, University of Padova, Padova, Italy; 2 Transplant Immunology Laboratory, Institute of Pediatric Research “Città della Speranza”, Padova, Italy; 3 Unit of Biostatistics, Epidemiology and Public Health, Department of Cardiac, Thoracic, Vascular Sciences and Public Health, University of Padova, Padova, Italy; 4 Laboratory of Immunopathology and Molecular Biology of the Kidney, Department of Women’s and Children’s Health, University of Padova, Padova, Italy; 5 Institute of Pediatric Research Città della Speranza, Padua, Italy; 6 Pediatric Nephrology, Department of Women’s and Children’s Health, Padova University Hospital, Padova, Italy; 7 Department of Surgery, Oncology and Gastroenterology (DiSCOG), University of Padova, Padova, Italy; 8 Transplant Immunology Unit, Padova University Hospital, Padova, Italy

**Keywords:** anti-ETAR, functional antibodies, G protein–coupled receptors, non-HLA antibodies, pediatric kidney transplantation

## Abstract

**Background:**

Antibody-mediated rejection remains the leading cause of kidney allograft failure in pediatric recipients. While non-HLA antibodies targeting G protein-coupled receptors, particularly anti-endothelin-1 type A receptor (ETAR) antibodies, have been implicated in allograft injury, their functional activity and independent pathogenic role in pediatric kidney transplantation remain poorly characterized.

**Methods:**

In this study involving 46 pediatric kidney transplant recipients, anti-ETAR antibodies were quantified and their functional activity assessed using a β-arrestin–based luminometric bioassay in ETAR-transfected CHO cells. Furthermore, IgG-induced migration of T-lymphocytes and NK-92 cells was evaluated by chemotaxis assay. Renal function was monitored longitudinally. Predictive models of eGFR decline between consecutive follow-up visits were developed using restricted cubic spline regression with bootstrap validation.

**Results:**

Patient-derived IgG exhibited concentration-dependent agonistic activity on ETAR, including samples with anti-ETAR IgG below the standard positivity threshold (17 U/mL). Moreover, IgG enhanced endothelin-1–induced receptor activation, independently of anti-ETAR antibody levels. IgG from pediatric transplant recipients with elevated anti-ETAR levels significantly increased T-lymphocyte and NK-92 cell migration, irrespective of the presence of anti-HLA antibodies. Anti-ETAR IgG levels were associated with immune cell migration and with the decline in eGFR (Spearman ρ = 0.52). In multivariable modeling, however, antibody concentration retained no validated predictive information after bootstrap optimism correction, whereas IgG-induced T-cell migration remained associated with eGFR decline after adjustment for time since transplantation and for HLA donor-specific antibodies (DSA).

**Conclusion:**

In pediatric kidney transplant recipients, anti-ETAR antibodies are functionally active irrespective of their levels, promoting receptor signaling and immune cell recruitment associated with graft function deterioration. These results suggest that functional assessment, particularly T-cell migration, may provide complementary information beyond antibody concentration and warrants further investigation as a potential approach to risk stratification.

## 1 Introduction

Kidney transplantation represents the ideal treatment option in children with end-stage renal disease as it is associated with better quality of life, increased survival, and reduced healthcare costs compared to dialysis (1, 2).

It is well known that the estimated half-life of pediatric transplanted kidneys is approximately 12–15 years (3). This implies that within such a period of time half of the transplanted patients will lose their graft, return to dialysis, and, when possible, will be listed for a second transplant. Intensive investigations have demonstrated that antibody-mediated rejection (AMR) currently accounts for the leading cause of kidney graft failure (4, 5). AMR is the consequence of a humoral immune response against the transplanted organ that is mediated by pre-existing or *de novo* anti-donor antibodies primarily directed against mismatched HLA molecules in the graft (6). It has been pointed out that other antibodies directed against non-HLA molecules may play a role in determining the fate of the graft (7).

These antibodies have different specificities and target molecules present on the membranes of endothelial cells of the graft, as well as in the basement membrane, the extracellular matrix, and intracellular compartments (8). The most extensively studied non-HLA antibodies in transplantation are those directed against G protein–coupled receptors (GPCRs), with particular attention to those targeting the angiotensin II receptor type 1 (AT1R). In this regard, several studies have demonstrated that the presence of anti-AT1R antibodies is associated with increased risk of kidney allograft rejection and reduced allograft survival (9), with a synergistic effect with donor-specific HLA antibodies on allograft injury (10). In addition, anti-AT1R antibodies appear to be strongly associated with the presence of another member of the anti-GPCR family, namely antibodies targeting the endothelin-1 type A receptor (ETAR). Similarly to anti-AT1R antibodies, anti-ETAR antibodies exert agonist activity on their target receptor, mediating vasoconstriction, oxidative stress, mesangial cell proliferation, renal parenchymal injury, loss of capillaries, cell proliferation, cell migration and differentiation, and renal and vascular inflammation (11).

In the context of pediatric transplantation, the effects mediated by anti-ETAR and anti-AT1R antibodies could be more prominent, as higher prevalence and higher levels of such antibodies have been found in pediatric recipients compared to adults (12).

While results to date are highly suggestive of a clinical role for non-HLA antibodies, some conflicting results have recently been reported, and several aspects still need clarification. First, their pathogenetic mechanism is not fully elucidated. Second, convincing data regarding anti-ETAR antibodies in pediatric kidney transplant recipients are scarce. Third, the lack of standardized positivity thresholds for anti-ETAR antibodies across studies has limited the ability to validate their clinical relevance and precludes the inclusion of anti-ETAR antibodies monitoring into routine kidney transplant practice.

To achieve a comprehensive immunological risk assessment in kidney transplantation, particularly in the pediatric setting, it is therefore crucial to refine the detection and increase the understanding of the functional activity of non-HLA antibodies, such as anti-ETAR antibodies, thereby potentially improving clinical management strategies.

## 2 Materials and methods

### 2.1 Patient population description and monitoring

This study was designed as a cross-sectional analysis, was conducted in accordance with the Declaration of Helsinki and was approved by the Ethics Committee of the Padua University Hospital. Written informed consent to participate was signed by all the patients included in the study. Serum samples were collected consecutively from pediatric kidney transplant recipients attending routine follow-up visits at the Pediatric Renal Transplant Center of Padua University Hospital between September 2021 and May 2022, irrespective of the time elapsed since transplantation. Therefore, each patient contributed a serum sample corresponding to the scheduled clinical visit during the enrollment period.

Pediatric recipients underwent assessment of allograft function, measurement of circulating anti- HLA- class I and Class II antibodies, anti-Endothelin type A (ETAR) receptor antibodies. In particular, following transplantation graft function was assessed using serum creatinine, estimated glomerular filtration rate (eGFR) calculated with the revised Schwartz formula, urinary protein excretion and blood urea levels. In this study, pediatric recipients were divided into different groups based on positivity for anti-HLA and anti-ETAR content. Of the 47 patients, one with an anti-ETAR concentration of 663 U/mL (approximately twice the next highest value in the cohort) was excluded as an extreme outlier before any analysis (**Supplementary Figure S1**), to prevent a single high-leverage observation from dominating the spline fits. All descriptive and modeling analyses therefore refer to the remaining 46 recipients. A sensitivity analysis re-incorporating this sample is reported in **Supplementary Table S1**. At our center, protocol kidney allograft biopsies are routinely performed at 6, 12, and 24 months after transplantation, whereas biopsies beyond 24 months are performed only when clinically indicated (for-cause biopsies). Consequently, histological data from biopsies performed either at the time or in temporal proximity to serum collection were available for only a limited number of patients (n = 15). Given this limited sample size, meaningful statistical analyses correlating histological findings with anti-ETAR antibody levels or functional assay results could not be performed.

### 2.2 Enzyme-Linked Immunosorbent Assay

Anti-ETAR antibodies were determined by ELISA using a 96‐well microtiter plates coated with ETAR extracts, in serum samples from pediatric patients collected at enrollment and stored at −80 °C. In addition to the serum samples collected at enrollment, longitudinal anti-ETAR measurements were available for all 46 patients at transplantation (T0) and at 6, 12, and 24 months post-transplantation. Serum samples (diluted 1:100), standards, and positive and negative controls were added to the plate and incubated at 4 °C for 2 hours. After 3 washing steps, plates were incubated for 60 minutes at room temperature with horseradish peroxidase‐labeled goat anti‐human immunoglobulin G, followed by incubation with 100 μL of the chromogenic substrate tetramethylbenzidine for 20 minutes. The absorbance of each well was analyzed at 450 nm with an ELISA reader (iEMS Reader MF Multiskan; Thermo). Each specimen was run in duplicate. Standard curves of anti-ETAR antibodies (2.5, 5, 10, 20, 40 U/mL) were included in each plate to enable the accurate quantification of antibodies in patient serum. Positivity for anti-ETAR antibodies was defined according to the threshold concentration (17 U/mL) provided by the manufacturer (CellTrend, Luckenwalde, Germany). Patients enrolled were classified as anti-ETAR-negative (<17 U/mL) or anti-ETAR-positive (≥17 U/mL) exclusively according to the antibody concentration measured in the enrollment sample, which was also used for IgG isolation and subsequent functional assays.

### 2.3 Isolation of IgG

IgG were isolated using the Melon™ gel IgG spin purification kit (Thermo Scientific, Waltham, MA, USA) according to the manufacturer’s instructions, with minor modifications. Briefly, sera collected from pediatric patients were diluted 1:10 in Melon Gel Purification Buffer. Melon gel slurry (500 µL) was dispensed into spin columns placed in a microcentrifuge tube, subsequently centrifuged at 4,000 x g for 1 min. The columns were washed twice with purification buffer, followed by a pulse centrifugation for 10 seconds. Diluted sera (500 µL) were loaded onto the columns and incubated at room temperature for 5 min with end-over-end mixing, then centrifuged at 4,000 x g for 1 min. To improve IgG purity, the flow-through fractions were reapplied to the columns and the procedure repeated twice. The obtained flowthroughs were pooled, and the eluted IgG were dialyzed against phosphate-buffered saline (PBS). To assess the purity of the purified samples, SDS-PAGE electrophoresis was performed, followed by Coomassie staining of the gels. IgG concentration was quantified using Bradford Protein Assay, according to the manufacturer’s instructions (AppliChem GmbH, Darmstadt, Germany).

### 2.4 Bioassay measuring functionally active anti-ETAR antibodies

The bioactivity of anti-ETAR antibodies was measured in ETAR-transfected CHO cells engineered to express both the ETAR and an intracellular β-arrestin signaling pathway that, when activated by agonists binding to ETAR, develop a luminescent signal (Eurofins DiscoverX, Celle L’Evescault, France). Briefly, CHO cells were dispensed into wells of 96-well culture plates and incubated for 48 hours. Ten microliters of the natural agonist endothelin-1(0.01-300nM, positive control) or purified IgG (from 0.25 to 5 mg/mL) were then added and incubated for 90 minutes, following the manufacturer’s recommendation. Detection reagents were then added, and the chemiluminescent signal was read on a luminometer (Spark^®^ Multimode Microplate Reader, Tecan, Switzerland). A standard dose response curve of endothelin-1 was obtained, and β-arrestin recruitment mediated by IgG was expressed as bioequivalents of endothelin-1 (nmol/L).

### 2.5 Cell cultures

PBMC and T-cell isolation. PBMCs were isolated from buffy coats collected from healthy blood donor volunteers. PBMCs were prepared by centrifugation through a density gradient on Hystopaque Lymphocyte Separation Medium (Sigma-Aldrich, Milan, Italy).

T-cells were isolated by magnetic-activated cell sorting using the Pan T-cell Isolation Kit (Voden, Meda, MB, Italy) according to the manufacturer’s protocol. The purity of the T-cells was higher than 98% as assessed by flow cytometry. Isolated T-cells were cultured overnight at a concentration of 2 million cells/mL in RPMI 1640 medium supplemented with 2% IgG-low fetal calf serum (FCS), 2 mM l-glutamine, 2 mM non-essential amino acids, 0.37 mM sodium pyruvate, 25 mM Hepes, 100 U/mL penicillin, and 100 µg/mL streptomycin (all from EuroClone^®^ Ltd, Paington, UK) and 50 nM β-mercaptoethanol (Sigma-Aldrich, Milan, Italy) in a humidified atmosphere with 5% CO2 at 37 °C. On the next day, cells were seeded at a concentration of 1 million cells/mL in anti-CD3/anti-CD28–coated well plates (10 and 5 μg/mL, respectively, coated overnight at 4 °C) (BioLegend, San Diego, USA) and cultured for 3 days in the presence of 1 μg/mL interleukin 2 (IL-2) (PeproTech, UK) under the conditions described above.

NK-92 cells. NK-92 cells, a well-established human NK cell line widely used to investigate NK cell migration and function, were employed as an *in vitro* model (13, 14). NK-92 cells were maintained in MEM-alpha media without ribonucleosides and deoxyribonucleosides supplemented with 12.5% fetal calf serum, 12.5% Horse serum, 2 mM glutamine, 1% v/v HEPES, 0.1 mM 2-Mercaptoethanol, 100 U/mL penicillin, 0.1 mg/mL streptomycin (all from EuroClone^®^ Ltd, Paington, UK), 100 U/mL IL-2 and sub-cultured twice per week.

### 2.6 Immunofluorescence analysis of cultured cells

Stimulated human T-cells or NK-92 cells were resuspended at a concentration of 5 × 10^6^ cells in 200 µL of culture medium and mixed 1:1 with 2% agarose in 1× PBS at 45 °C. After solidification, agarose cell pellets were fixed in 4% formaldehyde for up to 3 h at room temperature and processed for paraffin embedding using graded ethanol dehydration, xylene clearing, and paraffin infiltration at 58–62 °C. Paraffin blocks were sectioned at 3–5 µm and mounted on glass slides. Sections were deparaffinized, rehydrated, and subjected to heat-induced antigen retrieval in 10 mM sodium citrate buffer (pH 6.0) at ~100 °C for 20 min. After blocking with 10% fetal bovine serum (FBS) in PBS for 1 h at room temperature, slides were incubated overnight at 4 °C with rabbit primary anti-ETAR (Genetex, USA; GTX116034 antibodies diluted in 1% FBS in PBS.). After PBS washes, sections were incubated with FITC-conjugated anti-rabbit IgG secondary antibody for 1 h at room temperature in the dark. Negative controls were performed by omitting the primary antibody and incubating with the secondary antibody only. Slides were mounted with DAPI-containing mounting medium and images were acquired using a fluorescence microscope with identical acquisition settings for all conditions.

### 2.7 Migration assay

The migration of immune cells through permeable supports with 5 µm pore membranes was evaluated. In particular, T-cell migration was measured by seeding 200,000 cells into the 96-well plate inserts (upper chamber) in RPMI with 0.5% BSA (migration medium). The lower wells were filled with migration medium, with or without 0.5 mg/mL IgG collected from selected sera, in the presence of SDF-1α (PeproTech, UK) at 10 nM. The use of SDF-1α was established during preliminary assay optimization to provide a controlled inflammatory-like migratory environment and standardized chemotactic stimulus and to improve the sensitivity and reproducibility of the assay.

NK-92 cell migration was evaluated by seeding 600,000 cells into 24-well plate inserts (upper chamber). The lower well was filled in with or without 0.5 mg/mL IgG collected from selected sera. For both cellular assays, two-hour migration into the lower well was evaluated by measuring the fluorescence of resazurin dye (Sigma-Aldrich, Milan, Italy).

### 2.8 Statistical analyses

Continuous variables were reported as medians and interquartile ranges (IQRs) due to non-normal distributions, while categorical variables were summarized as frequencies and percentages. Differences between groups were assessed using the Wilcoxon rank-sum test for continuous variables and Fisher’s exact test for categorical variables, with Benjamini–Hochberg false-discovery-rate correction for group comparisons and for the Spearman correlation screens spanning multiple outcomes. Bivariate associations between continuous variables were assessed with Spearman’s rank correlation coefficient (ρ), reporting the test p-value and the BH-FDR q-value across the screen. To explore the functional form of the relationships between biomarkers and cellular responses, linear and smoothed scatterplot overlay (GAM, Generalized Additive Model) were used.

Each recipient contributed one serum sample collected during a routine follow-up visit (study visit) and one renal outcome measurement. The renal outcome was defined as the percentage decrease in estimated glomerular filtration rate (eGFR) between an earlier routine follow-up visit (reference visit) and the study visit. For 20 of the 40 recipients included in the model, the reference visit corresponded to the 24-month protocol visit. A value of 0 indicated no decrease in eGFR. The outcome reflected the overall change between the two visits and was not normalized to the duration of the interval, which ranged from 3 to 103 months (median, 24 months; **Supplementary Figure S3**). No significant association was observed between interval duration and the magnitude of eGFR decrease (Spearman’s ρ = 0.17, p = 0.28). The variables of interest were anti-ETAR antibody concentration, measured in the serum sample collected at the study visit, and IgG-induced T-cell migration, assessed using IgG purified from the same sample and expressed as a percentage relative to medium alone. The final model was adjusted for time since transplantation, defined as the number of months between transplantation and the study visit, and modeled using a restricted cubic spline.

The association between antibody concentration and cell migration was modeled using Ordinary Least Squares (OLS) regression. To account for potential non-linear biological dose-response relationships, restricted cubic splines (RCS) with 3 knots were employed. The confounding effect of time from transplant was evaluated and adjusted for in multivariable models. Similar modeling strategies were applied to Agonistic Activity, evaluating both linear and non-linear dependencies.

The final predictive model was adjusted for time elapsed since transplantation and modeled non-linearly using RCS to account for the variable rate of renal function decline over time. Internal validation was performed to assess the risk of overfitting using a nonparametric bootstrap with 500 resamples, with a fixed random seed, to estimate optimism in model performance measures. Calibration of the final model is reported in **Supplementary Figure S2**. To verify that the migration signal was not a proxy of the classical donor-specific antibody risk factor, the time-adjusted reference model was extended with HLA-DSA, entered (i) as a binary status and (ii) as a continuous burden defined as log1p of the maximum MFI across positive specificities, assigning zero value to HLA-DSA-negative patients. A sensitivity analysis was performed by re-fitting the same time-adjusted model on HLA-DSA-negative patients only. A nomogram summarizing the final model is provided in the **Supplementary Material** for illustrative purposes only (**Supplementary Figure S4**).

All statistical analyses were performed using R (version 4.5.2), with the `rms` package for spline regression and bootstrap internal validation, `gtsummary` for descriptive tables, and `ggplot2`/the `tidyverse` for data manipulation and visualization. A p-value < 0.05 was considered statistically significant.

## 3 Results

### 3.1 Study population and anti-ETAR levels

In this study, 46 pediatric renal transplant recipients were included. (flow of patients through the study is shown in **Supplementary Figure S1**; mean age ± SD: 8 ± 5 years, 12 females and 34 males). The clinical characteristics of the patients are reported in **Table 1**. All patients received basiliximab and steroids at induction and calcineurin inhibitors (tacrolimus or cyclosporine), mycophenolate mofetil and steroids as maintenance therapy. The source of the graft was a living donor in eleven cases and a deceased donor in the remaining thirty-five cases. Primary disease leading to end-stage renal disease was mainly represented by congenital abnormalities of the kidney and urinary tract (CAKUT).

**Table 1:** Patient and transplant characteristics according to anti-ETAR antibody levels.

Characteristics	ALL N = 46	<17 U/ml N = 22	>17 U/ml N = 24	p-value^1^	q-value^1^
Recipient age at transplantation (years)				0.079	0.393
Median [Q1-Q3]	8 [3-11]	9 [3-15]	6 [2-9]		
Mean (SD)	8 (5)	10 (6)	7 (4)		
Race				0.096	0.420
Caucasian	40 (87%)	17 (77%)	23 (96%)		
Non-Caucasian	6 (13%)	5 (23%)	1 (4.3%)		
Sex				0.514	0.823
Female	12 (26%)	7 (32%)	5 (21%)		
Male	34 (74%)	15 (68%)	19 (79%)		
Recipient’s blood group				0.743	0.977
0	16 (35%)	6 (27%)	10 (42%)		
A	22 (48%)	12 (55%)	10 (42%)		
B	7 (15%)	4 (18%)	3 (12%)		
AB	1 (2.%)	0 (0%)	1 (4%)		
Time on the waiting list (months)				0.574	0.873
Median [Q1-Q3]	11 [5-18]	11 [8-14]	16 [5-24]		
Mean (SD)	15 (12)	11 (5)	18 (16)		
Donor type				>0.999	>0.999
Deceased	35 (76%)	17 (77%)	18 (75%)		
Living	11 (24%)	5 (23%)	6 (25%)		
Previous transplants	4 (8.9%)	3 (14%)	1 (4.3%)	0.346	0.713
Number of HLA mismatches (0-6)				0.618	0.893
Median [Q1-Q3]	4.0 [3.0-5.0]	4.0 [3.5-5.0]	4.0 [3.0-5.0]		
Mean (SD)	4.0 (1.3)	4.2 (1.2)	3.9 (1.3)		
Time of follow-up (months) [Median Q1-Q3]				0.323	0.713
Median [Q1-Q3]	53 [37-78]	51 [23-87]	57 [39-77]		
Mean (SD)	59 (33)	55 (35)	63 (31)		
DSA positive patients	8	4	4		

Continuous variables are reported as median [Q1–Q3] and mean (SD); categorical variables as N (%). Group comparisons use the Wilcoxon rank-sum test for continuous variables and Fisher’s exact test for categorical variables, with Benjamini–Hochberg FDR-adjusted q-values.

Based on anti-ETAR antibody concentrations measured in the enrollment samples, 22 patients were classified as anti-ETAR-negative (<17 U/mL) and 24 as anti-ETAR-positive (≥17 U/mL). Additional anti-ETAR measurements obtained retrospectively and prospectively on longitudinal measurements, showed that antibody status remained stable, with patients maintaining their respective classification.

### 3.2 Functional activity of anti-ETAR antibodies from pediatric patients

#### 3.2.1 Agonistic activity of anti-ETAR antibodies

Increasing concentrations of purified IgG were assessed for their ability to activate ETAR using the assay described in the Methods. All IgG tested elicited a concentration-dependent chemiluminescent response, demonstrating activation of the receptor signaling. The highest signal was observed at an IgG concentration of 5 mg/mL (**Figure 1**), which was therefore used for all subsequent patient comparisons.

**Figure 1:**
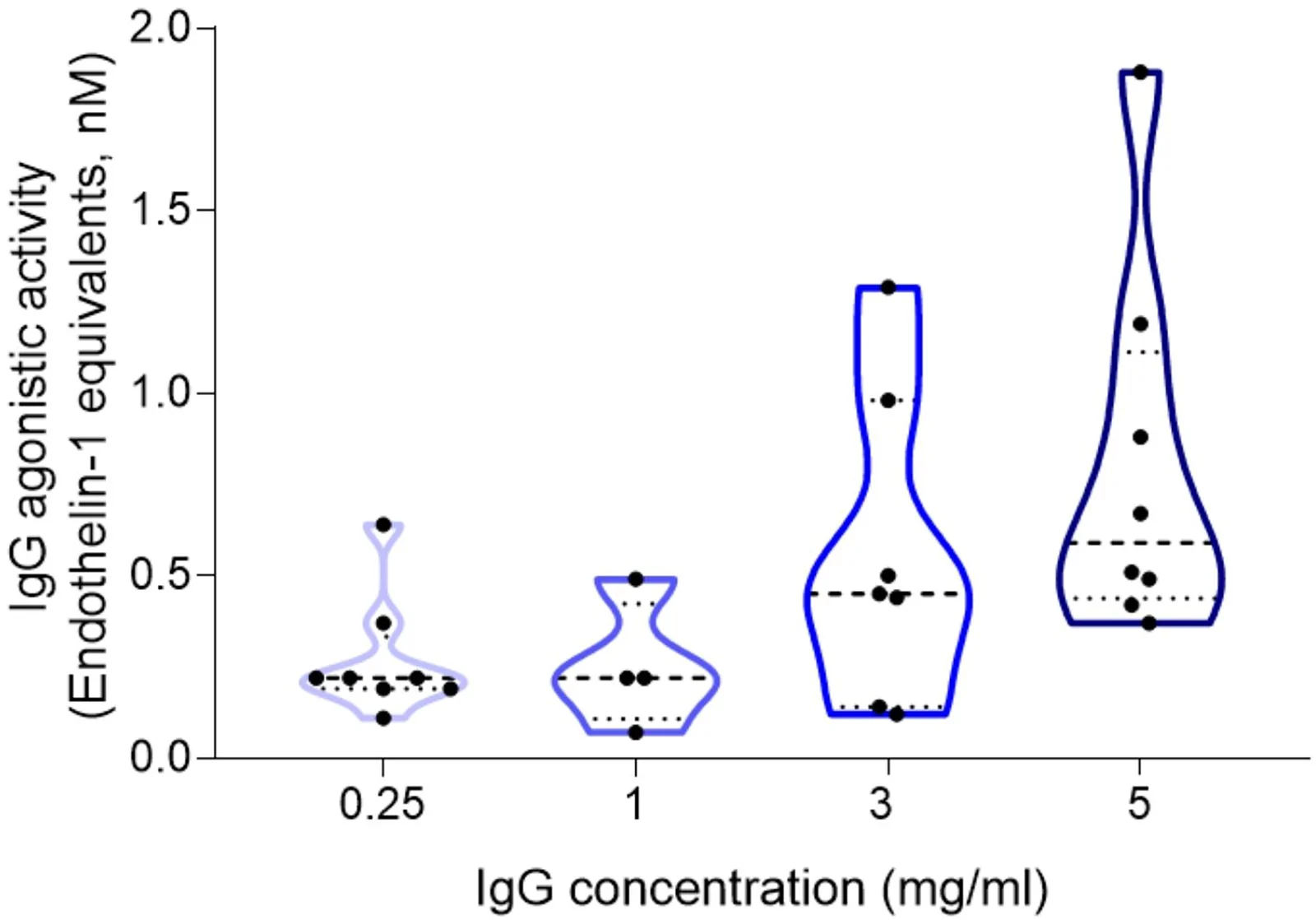
Concentration-dependent agonistic activity of patient-derived IgG on ETAR. Dose-response analysis of IgG agonistic activity on ETAR at increasing IgG concentrations (0.25, 1, 3, and 5 mg/mL) collected from a representative subset of pediatric kidney recipients. Data are expressed as Endothelin-1 equivalents (nM). Violin plots show the distribution of data with the median (dashed line) and the interquartile range (dotted lines). Individual data points are overlaid. Based on these results, an IgG concentration of 5 mg/mL was selected for subsequent experiments.

Although not statistically significant (p = 0.28), IgG isolated from patients with anti-ETAR values ≥ 17 U/mL exhibited a trend toward higher agonistic activity compared to those with levels <17 U/mL (median [Q1-Q3]: 0.67[0.49-0.82] vs 0.58 [0.37-0.71] nM) (**Figure 2**). Interestingly, measurable anti-ETAR-mediated agonistic activity was also detected after exposure to IgG from patients with anti-ETAR concentrations lower than 17 U/mL. The lowest anti-ETAR antibody concentration measured in a sample that produced a detectable agonistic response was 6.2 U/mL, corresponding to an agonistic activity of 0.5 nM endothelin-1 equivalents. The agonistic activity of the functionally active anti-ETAR antibodies did not correlate with ELISA-determined antibody levels (**Figure 3**).

**Figure 2:**
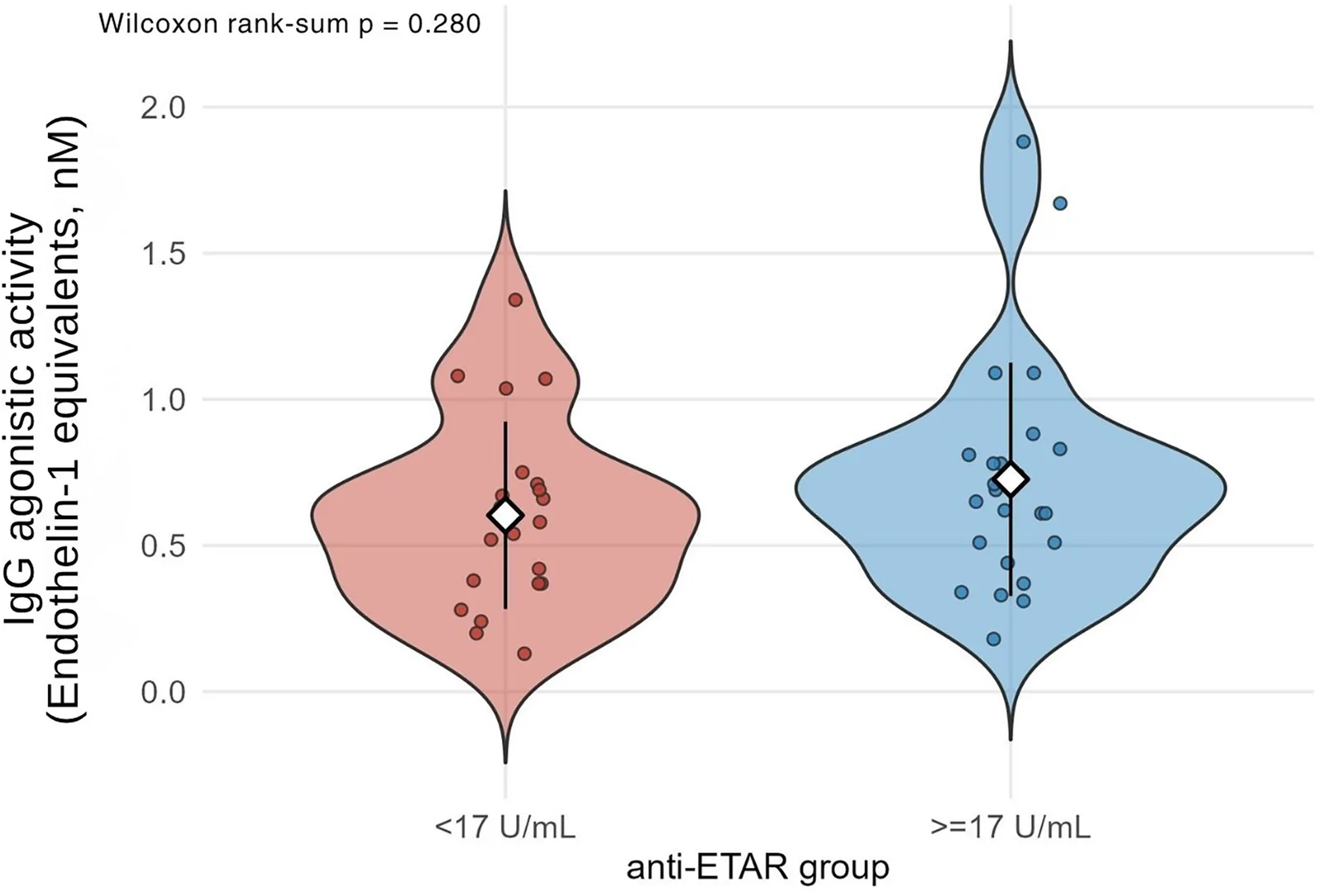
Agonistic activity of patient-derived IgG according to anti-ETAR antibody levels. Purified IgG (5 mg/mL) isolated from pediatric kidney transplant recipients (n = 45) were tested. Patients were stratified according to anti-ETAR antibody levels below (<17 U/mL) or above (≥17 U/mL). Agonistic activity was expressed as Endothelin-1 equivalents (nM). Violin plots show the distribution of the data with individual patient values overlaid. The white diamond indicates the mean. Groups were compared using the Wilcoxon rank-sum test.

**Figure 3:**
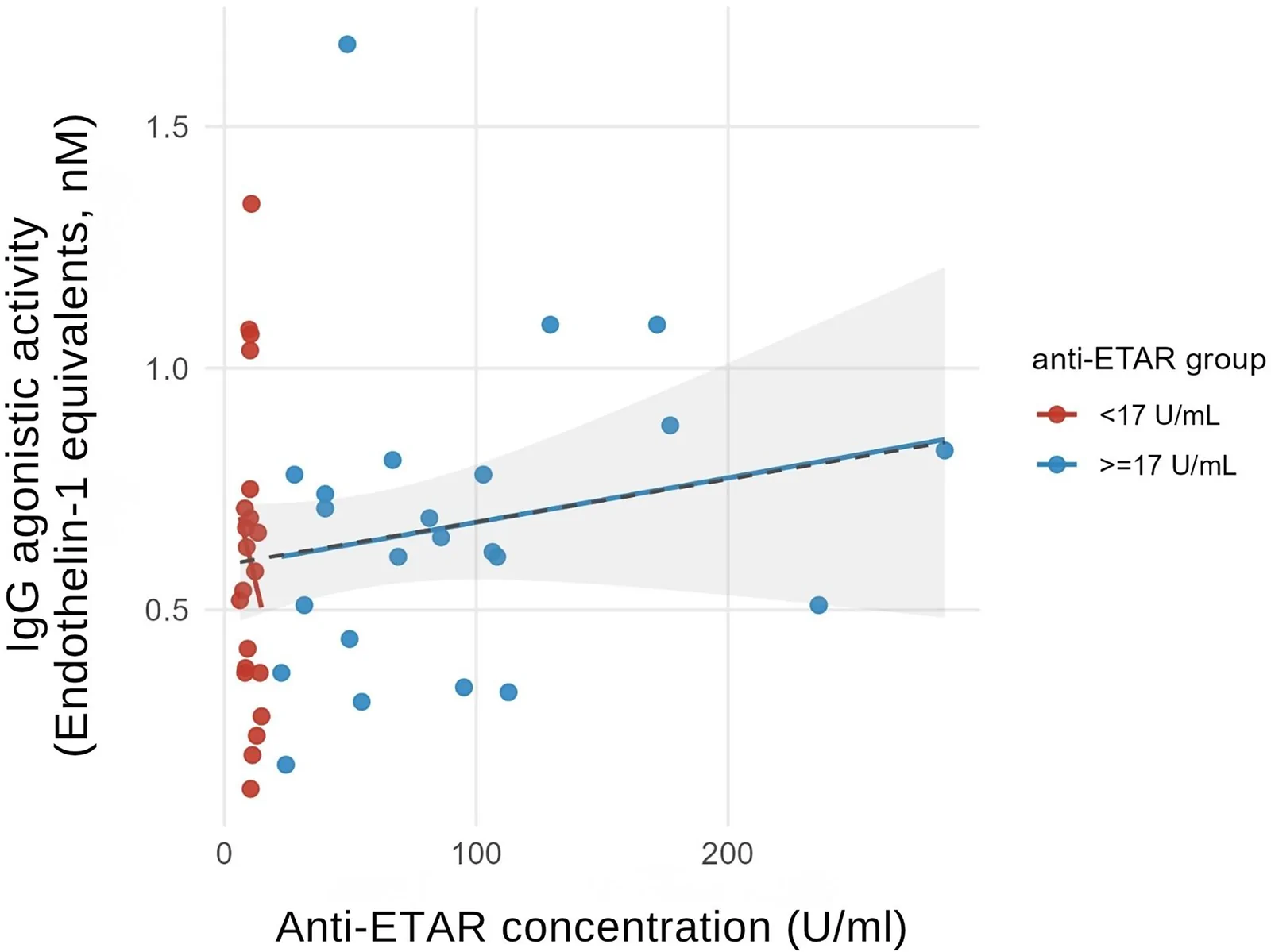
Correlation between anti-ETAR antibody levels (U/mL) and IgG agonistic activity. Samples are color-coded by group: anti-ETAR <17 U/mL (red), ≥17 U/mL (blue). The regression lines of the groups are reported.

To further investigate the potential interaction between anti-ETAR antibodies and endothelin-1, the endogenous ligand of the receptor, in a second set of experiments cells were exposed to IgG purified from the serum of two transplanted patients at the concentration of 0.25 mg/ml alone or in association with increasing concentration of endothelin-1 (from 0.01 to 300 nM).

The exposure to IgG collected from pediatric patients resulted in a consistent increase in endothelin-1–induced signaling across the entire concentration range (**Figure 4A**). Notably, this effect was also associated with an increase in the maximal response (Emax), indicating an enhancement of endothelin-1 efficacy. IgG were then screened and tested in association with endothelin-1 at the sub-threshold concentration of 0.03 nM. Under these conditions, the response of endothelin-1 was variably modulated by patient derived IgG (**Figures 4B, C**) Indeed, in a substantial proportion of samples, the presence of IgG enhanced the luminometric signal compared to endothelin-1 alone and exceeded the expected additive response calculated from the individual effects of IgG and endothelin-1. Overall, this enhancement was observed in 55% of samples with anti-ETAR levels <17 U/mL and in 52.4% of samples with anti-ETAR levels ≥17 U/mL. No significant difference was detected between the <17 U/mL and ≥17 U/mL anti-ETAR levels groups (Fisher’s exact test, p = 1.00; **Figure 4D**).

**Figure 4:**
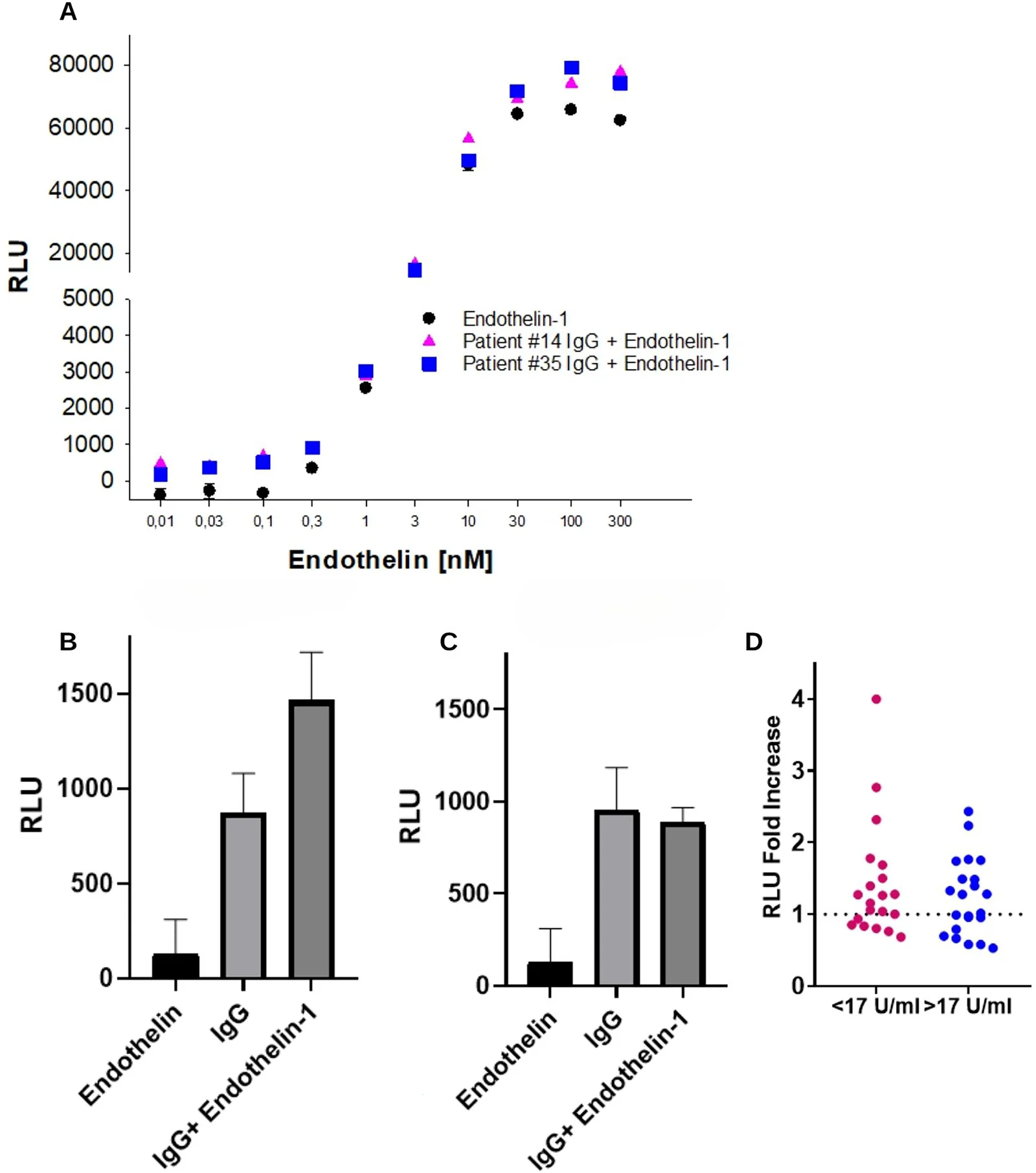
Patient-derived IgG enhance endothelin-1–induced ETAR receptor activation. **(A)** Concentration–response curves of Endothelin-1 in ETAR–expressing cells, measured using a β-arrestin–dependent luminometric assay, in the absence or presence of patient-derived IgG (0.25 mg/mL). Representative IgG samples from Patient #14 (anti-ETAR = 10.2 U/mL) and Patient #35 (anti-ETAR = 286 U/mL) enhanced receptor activation across the entire Endothelin-1 concentration range and increased the maximal response (Emax). Data are expressed as relative luminescence units (RLU). **(B, C)** Representative patient samples showing the heterogeneous effect of patient-derived IgG on endothelin-1–induced ETAR activation. **(B)** In one case, (anti-ETAR = 8.2 U/mL) IgG enhanced the response to endothelin-1, whereas in **(C)** (anti-ETAR = 10.4 U/mL) no enhancement was observed. Bars represent mean ± SD. **(D)** Distribution of fold increase in luminometric signal following IgG addition, expressed as the ratio between the observed combined response and the expected response obtained by the sum of IgG alone and Endothelin-1 alone at 0.03 nM. Each dot represents an individual patient sample, stratified according to anti-ETAR antibody cutoff of 17U/mL measured by ELISA.

#### 3.2.2 Chemotaxis of T-cells and NK cells

To analyze the impact of IgG on the migration of immune cells, chemotaxis assays were performed using isolated T-cells from healthy donors and NK-92 cells. Immunofluorescence analyses of these cells confirmed the presence of the ETAR receptor on the cell membrane (**Figures 5A, B**).

**Figure 5:**
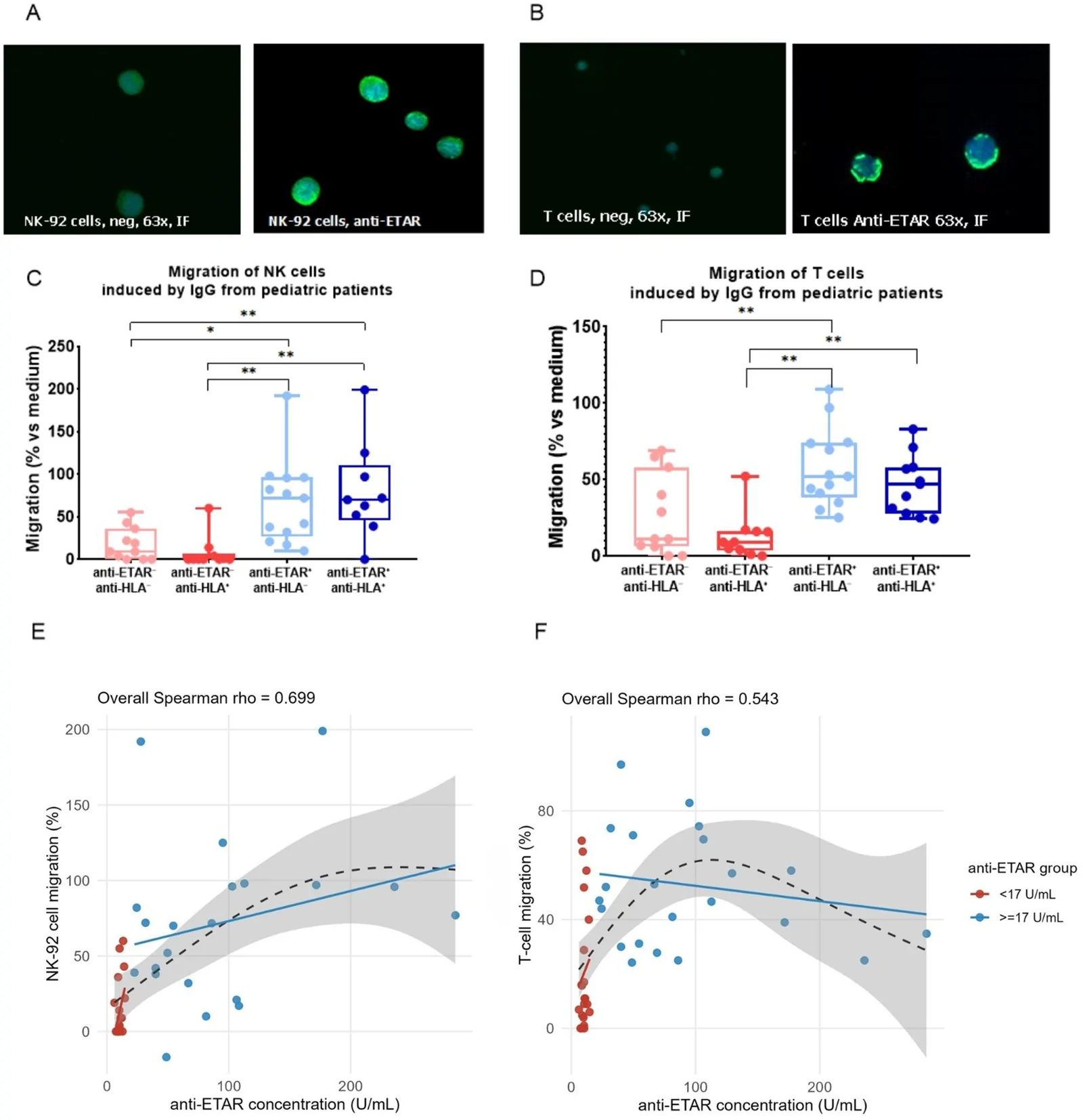
IgG promote NK-92 and T-cell migration in pediatric renal transplant patients. **(A, B)** Representative immunofluorescence images (63x) of NK-92 and T-cells stained with FITC-conjugated anti-ETAR antibodies. Left: negative control; Right: anti-ETAR positive staining (green). **(C)** Migration of NK cells induced by IgG from pediatric renal transplant patients, expressed as percentage versus medium. Patients were stratified into four groups: Group 1: anti-ETAR^-^/HLA^-^; Group 2: anti-ETAR^-^/HLA^+^; Group 3: anti-ETAR^+^/HLA^-^; Group 4: anti-ETAR^+^/HLA^+^. **(D)** Migration of T-cells induced by IgG from pediatric renal transplant patients, expressed as percentage versus medium. Group composition as in **(C)**. Statistical analysis was performed using one-way ANOVA followed by Tukey’s *post-hoc* test. **p<0.01. **(E, F)** Correlation between NK cell migration (%) and T-cell migration (%) vs anti-ETAR antibody concentration (U/mL). Samples are color-coded by group: anti-ETAR <17 U/mL (red) and ≥17 U/mL (blue). Dashed curve: overall GAM fit; solid lines: within-group linear fit.

In **Figures 5C, D**, the results of migration toward IgG are reported, grouped by anti-ETAR antibody levels and further sub-grouped according to the anti-HLA status. Compared to sera with low anti-ETAR antibody titers (< 17 U/mL) (Group 1 and 2), sera with the highest antibody titers (Group 3 and 4) were associated with increased NK-92 (**Figure 5C**) and T-cell migration (**Figure 5D**). Results were not influenced by the presence of anti-HLA IgG (Group 2 and 4).

When all patients were analyzed together, IgG-induced migration of both NK-92 and T-cells correlated with anti-ETAR antibody concentration (Spearman, p < 0.001 for both; **Figures 5E, F**). These overall associations were largely lost when the two anti-ETAR strata (<17 and ≥17 U/mL) were examined separately: three of the four within-stratum correlations were non-significant, the only exception being NK-92 migration in the <17 U/mL group (p = 0.042). This indicates that the association is driven mainly by the difference between the low- and high-concentration ranges rather than by a continuous dose-response relationship within each group.

The regression model between cell migration and antibody concentration confirms a dose-response relationship. The NK-92 cell model confirms a positive overall association (p=0.001) with anti-ETAR antibody levels, without a significant contribution (p=0.395) from transplantation time (**Figure 5E**). In the regression analysis of T-cell migration, antibody concentration appears to be the primary driver of cell recruitment (p<0.0001). Still, the non-linear spline shape suggests that, beyond a threshold, the biological effect reaches a plateau, after rising steeply up to an anti-ETAR antibody concentrations of approximately 100 U/mL. Subsequently, the chemotactic effect flattens or even attenuates at higher concentrations; given the limited number of observations above 150 U/mL, the apparent decline at the upper end should be interpreted with caution and may partly reflect data sparsity (**Figure 5F**). After adjustment for time since transplantation (non-linear, restricted cubic splines), anti-ETAR antibody concentration retained its association with cellular migration, indicating that the chemotactic capacity of the IgG fraction is not merely a consequence of overall graft exposure. Indeed, at any time point following transplantation, higher anti-ETAR antibody concentrations are still associated with greater immune-cell migration.

### 3.3 Relationship between anti-ETAR and kidney functionality

Graft function parameters, including blood creatinine, eGFR, and urea, were evaluated at the time of sample collection and compared with those from the previous follow-up visit. Renal function was stratified according to the levels of anti-ETAR antibodies (**Figure 6**). Compared with pediatric patients with anti-ETAR antibody levels under 17 U/mL, patients with anti-ETAR antibody levels exceeding such a cutoff showed a trend toward greater deterioration of serum creatinine and urea, and a nominally significant decrease in eGFR (Wilcoxon rank-sum p = 0.27, 0.20, and 0.013, respectively; **Figure 6**). When anti-ETAR IgG were analyzed as a continuous variable, Spearman rank correlations confirmed and strengthened the eGFR findings (ρ = 0.52, p < 0.001, q = 0.005; the positive sign indicates that higher antibody levels correspond to a larger percentage decrease in eGFR, corresponding to worse graft function) and brought the urea association to nominal significance (ρ = 0.38, p = 0.016, q = 0.062), consistent with a graded relationship that is partially diluted by the binary 17 U/mL cutoff.

**Figure 6:**
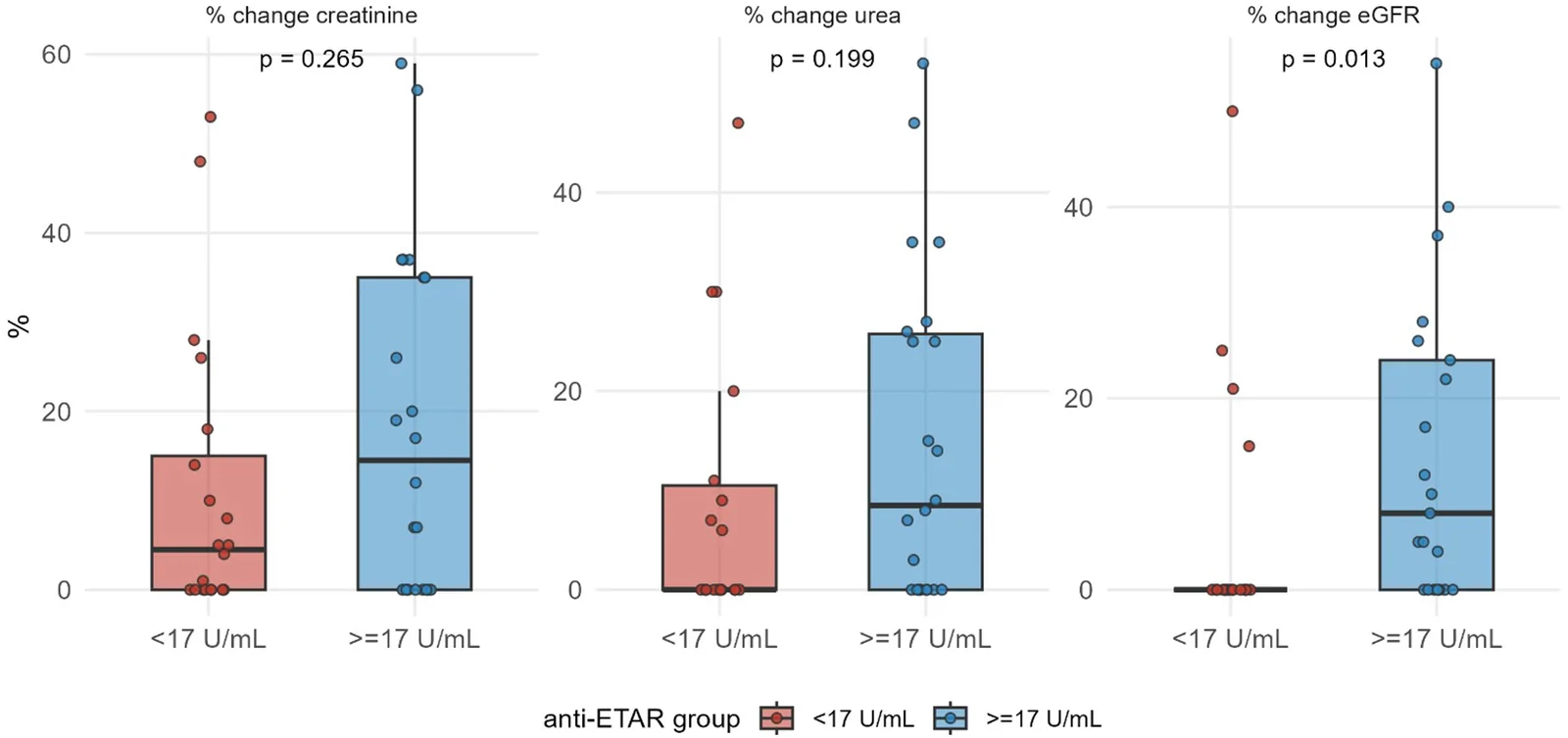
Renal function parameters in pediatric renal transplant patients stratified by anti-ETAR antibody levels. Box plots showing the percentage change (Δ%) in creatinine (left), urea (middle), and percentage decrease in estimated glomerular filtration rate (eGFR, right) relative to the earlier follow-up visit (reference visit), in patients with anti-ETAR antibody levels below (<17 U/mL, red) or above (≥17 U/mL, blue) the test positivity threshold. Statistical analysis was performed using the Wilcoxon rank-sum test (equivalent to the Mann–Whitney U test).

In addition, there was a highly significant positive correlation between the functional capability of anti-ETAR IgG antibodies to recruit immune cells, mainly T-cell, and eGFR decline (Spearman Rho value: 0.54, p<0.001 and Spearman Rho value:0.41, p<0.01, for T-cell migration and NK-migration, respectively).

A comprehensive statistical regression analysis was performed to develop and validate a predictive model of eGFR decline. The model of eGFR decline was fitted using data from the 40 recipients for whom the eGFR change, as defined in the Methods, was available. The six recipients excluded due to missing outcome data did not differ significantly from those included in terms of anti-ETAR antibody concentrations (Wilcoxon test, p = 0.72). Patient flow through the study is illustrated in **Supplementary Figure S1**. Restricted cubic splines (RCS) with 3 knots were used for all continuous predictors, including time since transplantation, to accommodate non-linear effects, and internal validation used a non-parametric bootstrap with B = 500 resamples to estimate optimism in model performance.

Anti-ETAR IgG concentration alone explained approximately 15% of the variability in eGFR decline (apparent R² = 0.15) and, after bootstrap correction for over-optimistic in-sample performance was removed, carried essentially no out-of-sample predictive information (optimism-corrected R² ≈ 0.01, 95% CI [−0.84, 0.29]). Replacing concentration with the functional readout of anti-ETAR IgG-induced T-cell migration substantially improved performance (apparent R² = 0.33, with borderline non-linearity, p = 0.053). The combined model with both anti-ETAR IgG concentration and T-cell migration produced no real gain (apparent R² = 0.37). Indeed, anti-ETAR IgG concentration becomes entirely non-significant, and Variance Inflation Factors above 35 for the concentration terms confirmed severe collinearity between anti-ETAR IgG concentration and T-cell migration, indicating that the two share most of their prognostic information for eGFR decline. In the final model, by contrast, all variance inflation factors were below the conventional threshold of 10 (8.2 and 8.7 for the two spline terms of T-cell migration, 9.4 and 9.0 for those of follow-up time), indicating that the two retained predictors do not carry redundant information.

The final model integrates T-cell migration (active immunological component) and time since transplantation (also RCS). Both contribute independently (T-cell migration p < 0.001; follow-up time p ≈ 0.017), the non-linear component of T-cell migration now reaches conventional significance, and the model attains an apparent R² of 0.47, with optimism-corrected R² = 0.29 and calibration slope 0.90 (**Figure 7**; calibration plot in **Supplementary Figure S2**). The fitted curve is flat at low migration values and rises at higher ones; with the present sample size, however, the position of this change in slope cannot be estimated with useful precision, and no biological threshold should be inferred from it. Based on the final model, a nomogram was developed to facilitate clinical interpretation (**Supplementary Figure S4**). However, this nomogram is provided for illustrative purposes only and should not be considered a validated clinical prediction tool or used for individual patient risk estimation until externally validated in independent cohorts.

**Figure 7:**
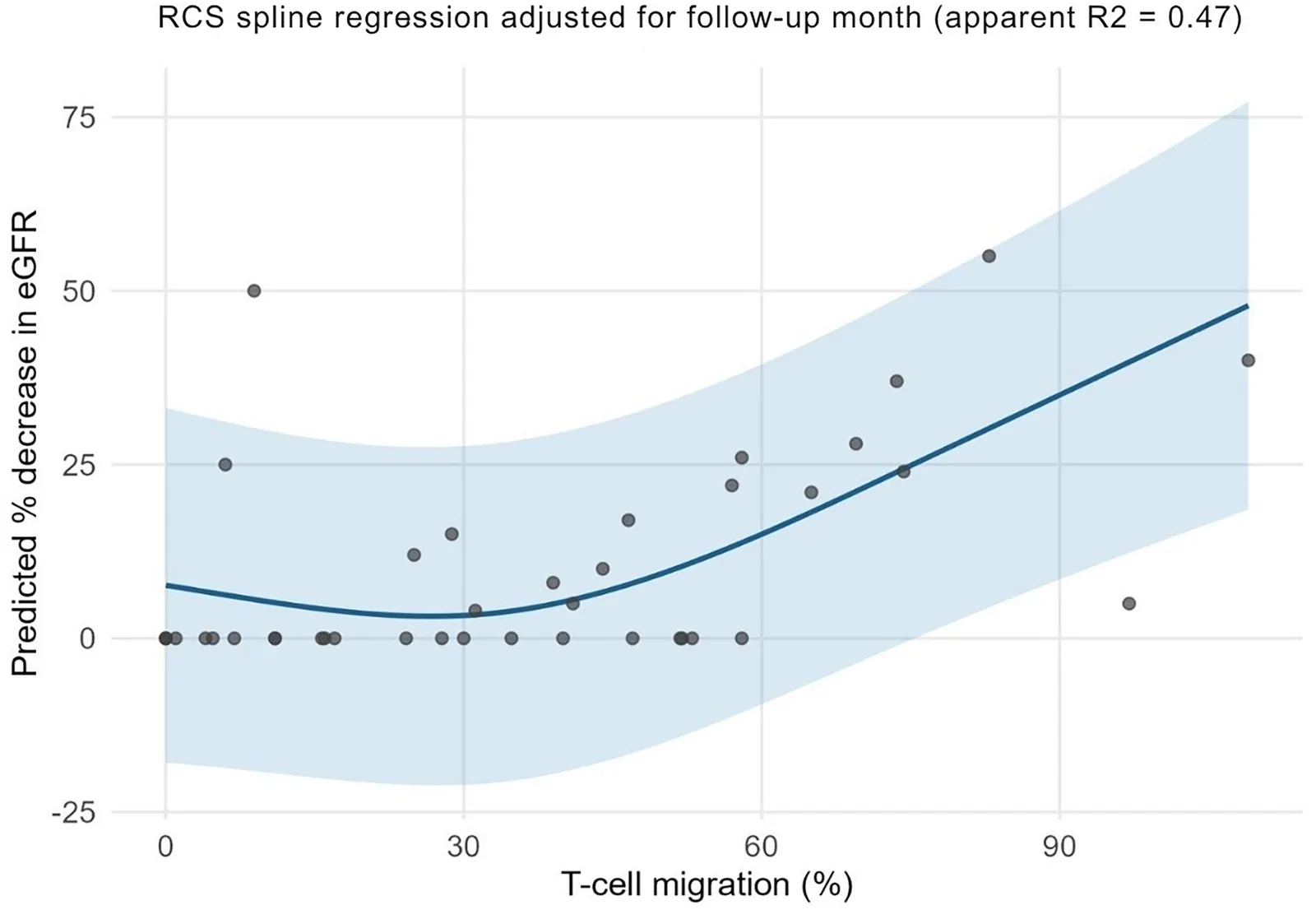
Prediction of eGFR decline by T-cell migration in pediatric renal transplant patients. Multivariable bootstrap-validated model for the percentage decrease in eGFR, including T-cell migration and time since transplantation (months from transplantation to the study visit; “follow-up month” in the panel title) as restricted cubic splines with 3 knots (n = 40; apparent R² = 0.47; optimism-corrected R² = 0.29 after B = 500 bootstrap resamples; calibration slope = 0.90). Shaded band: 95% confidence interval.

#### 3.3.1 Adjustment and sensitivity analyses for donor-specific HLA antibodies

The cohort included 8 HLA-DSA-positive patients. HLA-DSA were distributed similarly across the two anti-ETAR IgG groups (4 vs 4; Fisher’s exact test p = 1.00), so the difference in renal outcomes between anti-ETAR IgG groups cannot be attributed to imbalanced HLA-DSA exposure. Our final adjusted model is therefore obtained by extending the time-adjusted reference model above with HLA-DSA, both as a binary status (apparent R² = 0.58, HLA-DSA p = 0.004) and as the continuous burden log1p (MFI of the strongest DSA) (apparent R² = 0.56, HLA-DSA p = 0.009). T-cell migration retained a highly significant nonlinear effect (p < 0.001 overall, p ≈ 0.007 for the nonlinear sub-component in both models). A sensitivity analysis restricted to HLA-DSA-negative patients reproduced the migration-time association (apparent R² = 0.54; T-cell migration, p < 0.001). Together, these results indicate that the chemotactic readout provides prognostic information that is compatible with an additive contribution beyond classical HLA-DSA status. On the other hand, the limited number of HLA-DSA-positive patients (n = 8) precludes a confident assertion of full independence.

## 4 Discussion

This study provides new insights into the possible role of non-HLA antibodies in pediatric kidney transplantation, focusing on the functional activity of antibodies targeting the endothelin-1 type A receptor (ETAR). In this context, previous clinical studies have demonstrated a positive correlation of antibodies targeting ETAR, often in association with anti-AT1R antibodies, with decreased allograft survival, as well as possibly deleterious induction of *de novo* anti-HLA-donor specific antibodies (15, 16). However, whether screening for such GPCR-targeting antibodies provides additional value to current clinical practice for the management of solid organ recipients and risk stratification remains under discussion. Indeed, recent reports have challenged the value of monitoring anti-AT1R and ETAR antibodies in providing additional information beyond standard HLA-DSA assessment to predict allograft rejection (17, 18). In the prospective multicenter EU-TRAIN study (17), anti-ETAR antibody levels did not provide clinically useful discrimination of kidney allograft rejection. Similarly, Harmacek et al. (18) found no significant differences in non-HLA antibody concentrations across histologic rejection categories or biopsy-based molecular rejection scores. Although higher anti-ETAR antibody concentrations were observed in patients with molecular AMR, it remains unclear whether these antibodies mechanistically contribute to allograft injury or, instead, arise as a consequence of HLA antibody-mediated AMR.

Detection of this particular class of functional antibodies has moved from initial cell-based assays using spontaneously beating cultured rat cardiomyocytes to a standardized ELISA method using receptor-coated plastic plates in their native conformation (8). However, antibodies detected with such assays may bind to epitopes that are not necessarily involved in receptor activation.

In the current study, using a commercially available luminometric assay with CHO cells overexpressing ETAR, we have demonstrated that, beyond simple detection by ELISA, IgG collected from pediatric patients possess an agonistic biological activity that specifically activates receptor signaling. In addition, these IgG can increase immune cell migration. These findings provide functional evidence that the biological activity of patient-derived IgG may not be fully captured by ELISA-measured antibody concentration and highlight a potential mechanistic link between anti-ETAR antibody responses and immune-mediated injury in the transplanted kidney.

Our data confirm that functionally active anti-ETAR antibodies are frequently present in pediatric renal transplant recipients. Importantly, receptor activation was observed even in sera with anti-ETAR antibody concentrations below the manufacturer’s positivity threshold. Importantly, thresholds for defining anti-ETAR antibody positivity are not uniform across studies, with different cutoff values being reported. According to the manufacturer’s assay interpretation, antibody concentrations between 10 and 17 U/mL fall within an at-risk/gray-zone range, whereas concentrations ≥17 U/mL are considered positive. Indeed, in this study, antibody levels above 17 U/mL were always associated with measurable agonistic activity on the target receptor, although the magnitude of receptor activation was not proportional to the ELISA titer. Notably, receptor activity was also observed in several samples with antibody concentrations within the 10–17 U/mL at-risk/gray-zone range and, in some cases, at concentrations below 10 U/mL.

These findings suggest that conventional serological assays may underestimate the biological relevance of low-titer anti-ETAR antibodies and that the addition of functional testing could provide a more accurate measure of their pathogenic potential.

A key finding of this study is the enhancement of ETAR signaling when patient-derived IgG were combined with endothelin-1. In a substantial proportion of samples, the combined response exceeded the expected additive response calculated from the individual effects of IgG and endothelin-1, suggesting a supra-additive functional interaction between patient-derived IgG and the endogenous ligand. Such cooperative interactions varied across patients and were not associated with anti-ETAR antibody levels. Although this finding is consistent with an allosteric mechanism, the precise mechanism of receptor engagement remains to be established. However, alternative explanations, including receptor clustering, changes in receptor trafficking or internalization, or other forms of agonistic interaction, cannot be excluded. The observed heterogeneity across patients likely reflects differences in antibody affinity, epitope specificity, or functional subclass composition, rather than total antibody concentration. Indeed, it has been reported that functional non-HLA antibodies can recognize a specific domain of the receptor’s second extracellular loop (19). However, the antibody response is polyclonal, and we cannot rule out at this stage that antibodies recognizing GPCR domains other than loop 2 may arise. Their presence could be associated with activation or inhibition of such receptors or, as in some cases, stabilize active conformations of ETAR. Such *in vitro* observed effect could translate *in vivo* into sustained vasoconstriction, endothelial activation, and pro-inflammatory signaling, all mechanisms known to contribute to chronic allograft vasculopathy and interstitial fibrosis (11). On the other hand, such hypotheses will require additional experimental investigations.

The possibility that subclass functional composition may also be relevant, is supported by previous studies showing that the IgG subclass of non-HLA receptor-directed antibodies may influence their clinical and functional effects. In particular, anti-angiotensin II type 1 receptor antibodies of the IgG3 subclass have been reported to provide greater predictive value for antibody-mediated rejection than total anti-AT1R IgG1–IgG4 levels (20). Because patient-derived IgG were purified using Melon Gel without subclass-specific separation, the contribution of individual IgG subclasses could not be assessed in the present study. Further studies specifically characterizing anti-ETAR IgG subclasses will therefore be required to determine whether subclass composition contributes to the heterogeneous functional responses observed in our cohort.

The chemotaxis studies further support a functional link between anti-ETAR antibodies and immune activation. IgG isolated from pediatric recipients with high anti-ETAR levels significantly enhanced the migration of T lymphocytes and NK-92 cells, regardless of anti-HLA antibody presence. Similar migratory effects mediated by anti-ETAR and anti-AT1R antibodies have previously been reported in autoimmune diseases such as systemic sclerosis (21). In the pediatric transplantation context, given the agonistic activity observed in the ETAR-specific bioassay and the expression of ETAR on both T cells and NK-92 cells, ETAR activation represents one plausible mechanism underlying this effect. However, the contribution of additional signaling pathways or other antibody specificities cannot be excluded and warrants further investigation. Therefore, future studies employing pharmacological inhibition and complementary mechanistic approaches will be required to confirm the specific role of ETAR signaling in this process.

The observed correlation between antibody levels and migratory activity underscores the immunomodulatory capacity of these antibodies. Importantly, recent evidence has highlighted the critical role of intragraft expansion of T-cell clones in antibody-mediated kidney transplant rejection, supporting the concept that recruitment and local activation of T-cells are central drivers of graft injury (22). In this context, the observed association between anti-ETAR-positive IgG and enhanced T-cell migration may represent a biologically relevant mechanism contributing to allograft damage. Altogether, the independence of the observed effects from the presence anti-HLA antibodies suggests that ETAR-targeting antibodies may represent a distinct, non-HLA–driven pathway of allograft injury. This is particularly relevant in pediatric transplantation where non-HLA antibodies have been reported at higher prevalence and levels compared to adults and may exert more pronounced biological effects (12).

This study integrates functional immunological data with clinical outcomes. While anti-ETAR antibody levels showed only limited predictive value for graft dysfunction, functional assays —particularly T-cell migration—were associated with decline in renal function.

Notably, T-cell migratory activity was associated with a significantly greater eGFR decline compared to antibody concentration alone and remained the strongest predictor in multivariate models adjusted for time since transplantation. These findings indicate that functional properties of antibodies may represent a more accurate biomarker of graft injury than their mere presence or concentration.

We have therefore identified a composite risk profile in which eGFR decline is associated with both the magnitude of the cellular response (T-cell migration) and the time elapsed since transplantation.

Although the present study does not definitively establish a causal or mechanistic relationship, these findings are consistent with the hypothesis that enhanced immune-cell activation and migration may contribute to kidney injury. In particular, the amount of antibody appears to be relevant only to the extent that it translates into increased cell migration.

Routine testing protocols and a defined cut-off for these anti-GPCR functional antibodies are still lacking. Our data highlight the need to incorporate high-throughput assays into current post-transplant surveillance to quantify not only the presence of these antibodies but also their functional activity, thereby refining immunologic risk assessment and ultimately improving long-term graft outcomes in children.

Several limitations of our study should however be acknowledged. First, our anti-ETAR antibody measurements were performed in a single center although the results of some of the samples were confirmed by another laboratory. Second, the functional assays were performed *in vitro* using engineered or immortalized cell systems that may not fully recapitulate the complexity of the *in vivo* environment. In particular, the use of CHO cells overexpressing ETAR may increase assay sensitivity and potentially amplify receptor responses compared with native cells. For the migration studies, two complementary models were used: NK-92 cells as a standardized model of NK-cell function and primary T cells from healthy donors as a more physiologically relevant human T-cell model. Nevertheless, neither model fully reproduces the cellular and molecular complexity of the renal allograft microenvironment. Third, the cohort size limits the statistical power to detect moderate associations. With this sample size, our analyses can reliably detect strong effects (ρ ≳ 0.5 or R² ≳ 0.3) but are underpowered for moderate ones, so null findings (e.g., on agonistic activity vs absolute eGFR and on endothelin-1 potentiation across anti-ETAR groups) should be interpreted as absence of evidence rather than evidence of absence. Internal bootstrap validation reduces optimism bias but does not replace external validation, which remains the natural next step. Finally, only a small number of anti-ETAR positive patients had anti-HLA DSA precluding the analysis of the effects related to the presence of both antibodies. Moreover, histological data were available for only a limited number of patients, precluding meaningful correlation of anti-ETAR functional activity with graft histology.

In conclusion, our findings demonstrate that anti-ETAR antibodies in pediatric kidney transplant recipients are functionally active, enhancing receptor signaling and immune cell migration, which is associated with reduced graft function. These data are consistent with a pathogenic role for non-HLA antibodies in pediatric allograft injury, a hypothesis that remains to be confirmed in longitudinal studies with external validation.

Altogether, our data suggest that incorporating high-throughput assays into the current post-transplant surveillance to quantify not only the presence but also the functional activity of anti-ETAR antibodies, may help refine immunologic risk assessment and ultimately improve long-term graft outcomes in children.

## Data Availability

The raw data supporting the conclusions of this article will be made available by the authors, without undue reservation.
