# Supplementary material for: Functional activity of anti-endothelin type A receptor antibodies as a predictive tool of allograft injury in pediatric kidney transplantation
Source: Front Immunol. 2026 Oct 22;17:1925567. doi: 10.3389/fimmu.2026.1925567 (PMC13735602; doi:10.3389/fimmu.2026.1925567)
Supplement: Supplementary file 1 [file DataSheet1.pdf]

Supplementary Material

Functional activity of anti-Endothelin Type A Receptor antibodies as a predictive tool of allograft injury in pediatric kidney transplantation

Vadori M, Demirkilic E, Lanera C, Antoniello B, Negrisolo S, Vianello E, Di Bella C, Benetti E, Cozzi E

* Correspondence: marta.vadori@unipd.it

Contents

Supplementary Figure S1. Flow of patients through the study.

Supplementary Figure S2. Bootstrap-validated calibration of the final model.

Supplementary Figure S3. Distribution of the interval between the two eGFR measurements.

Supplementary Figure S4. Nomogram derived from the multivariable model.

Supplementary Table S1. Sensitivity analysis for the 600 U/mL exclusion.

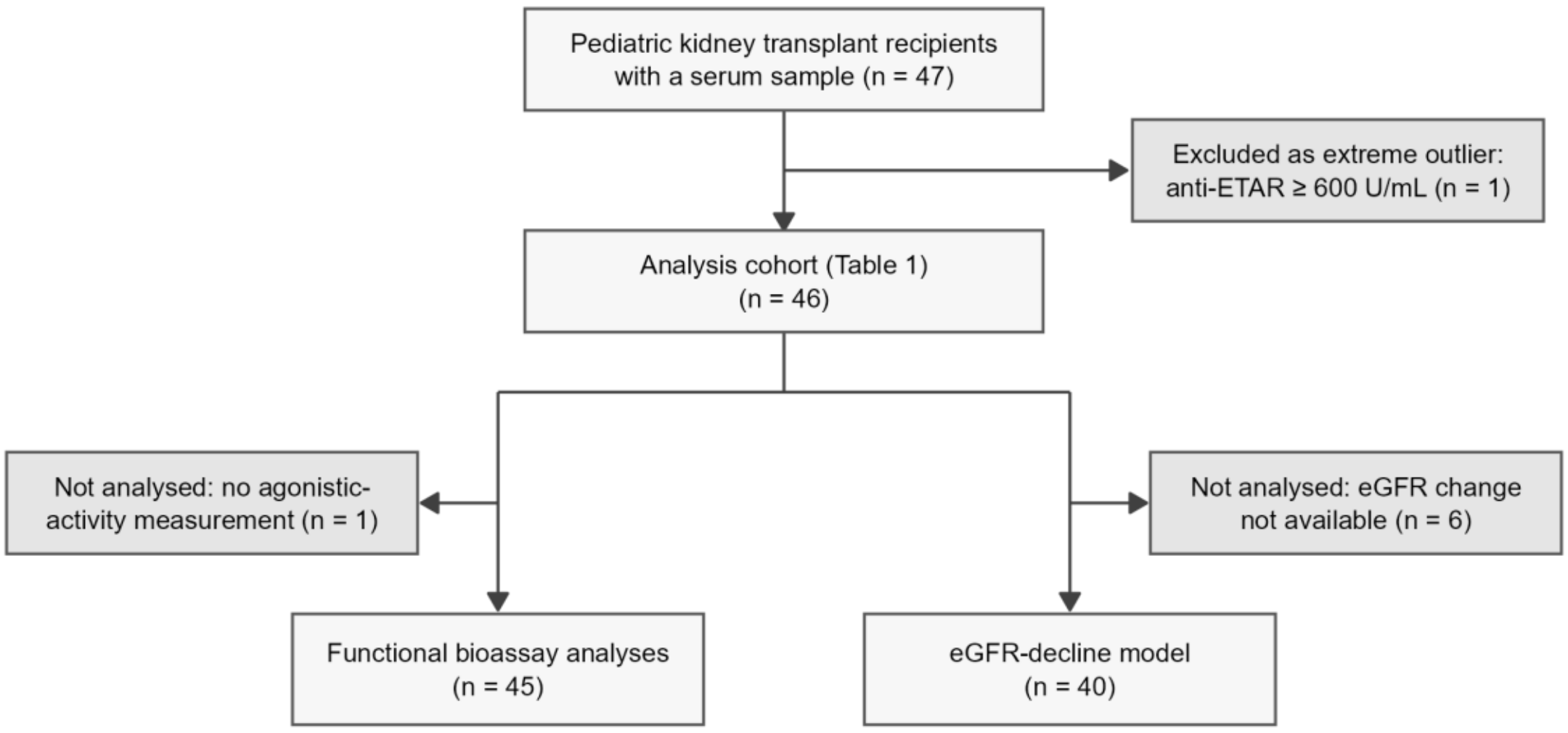

Supplementary Figure S1. Flow of patients through the study. Of the 47 serum samples processed, one was excluded as an extreme outlier (anti-ETAR ≥ 600 U/mL). The analysis cohort comprises 46 recipients; 45 had a measurable agonistic activity and 40 had an eGFR change available for the modelling of renal decline.

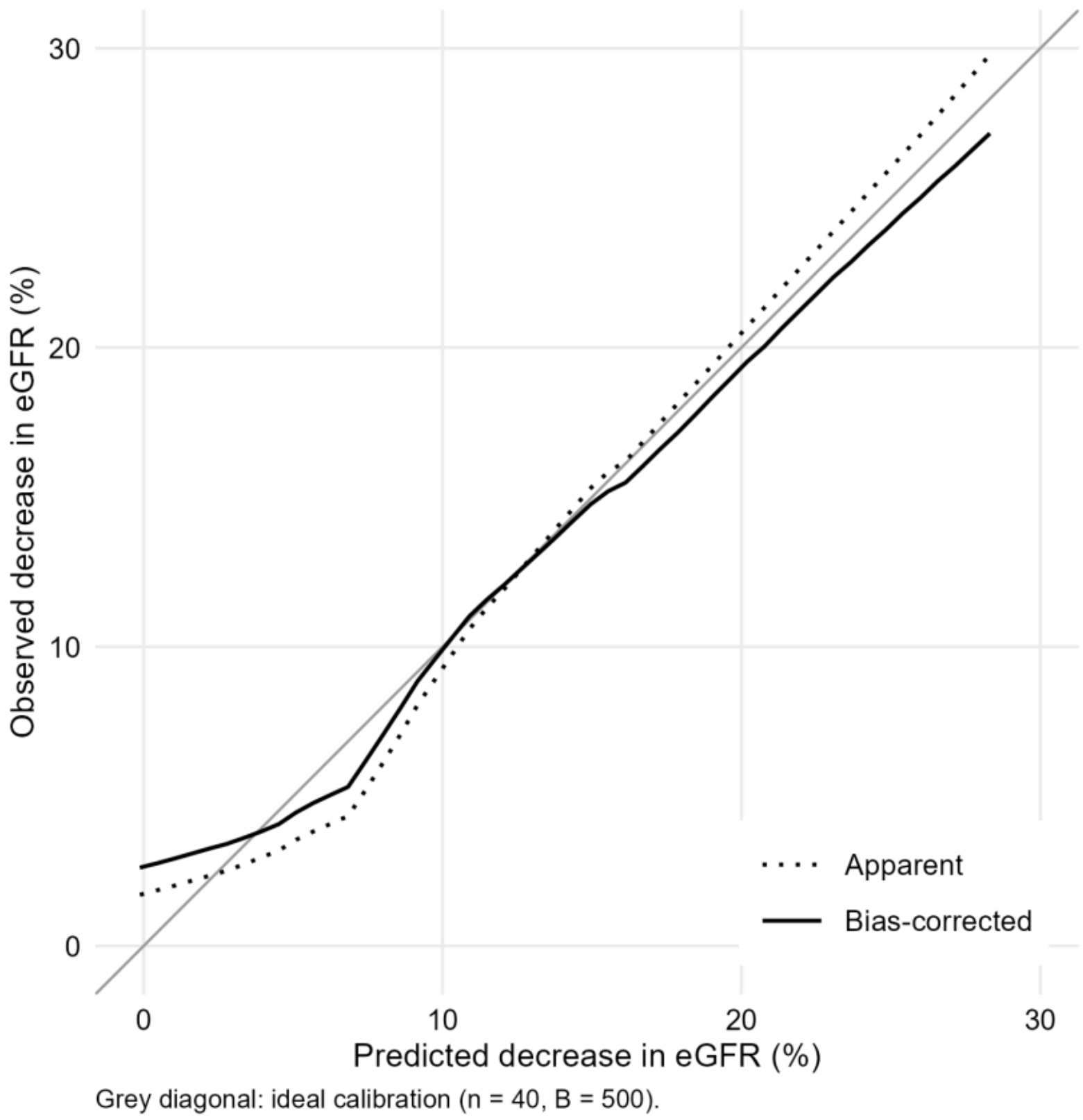

Supplementary Figure S2. Bootstrap-validated calibration of the final model for the percentage decrease in eGFR (n = 40, B = 500 resamples; calibration slope 0.90). The grey diagonal is ideal calibration.

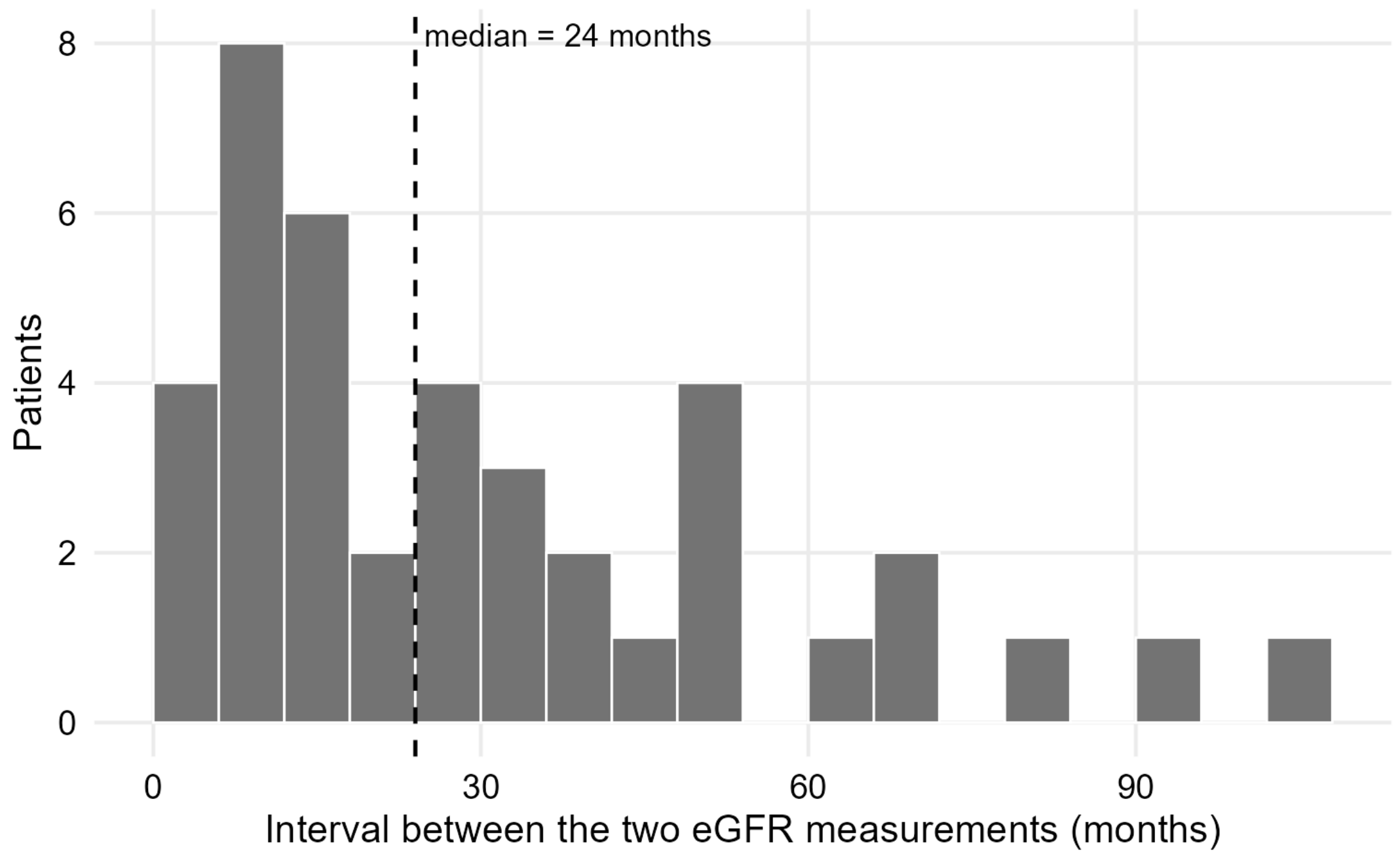

Supplementary Figure S3. Distribution of the interval between the two eGFR measurements used to compute the outcome (n = 40; range 3 to 103 months, median 24).

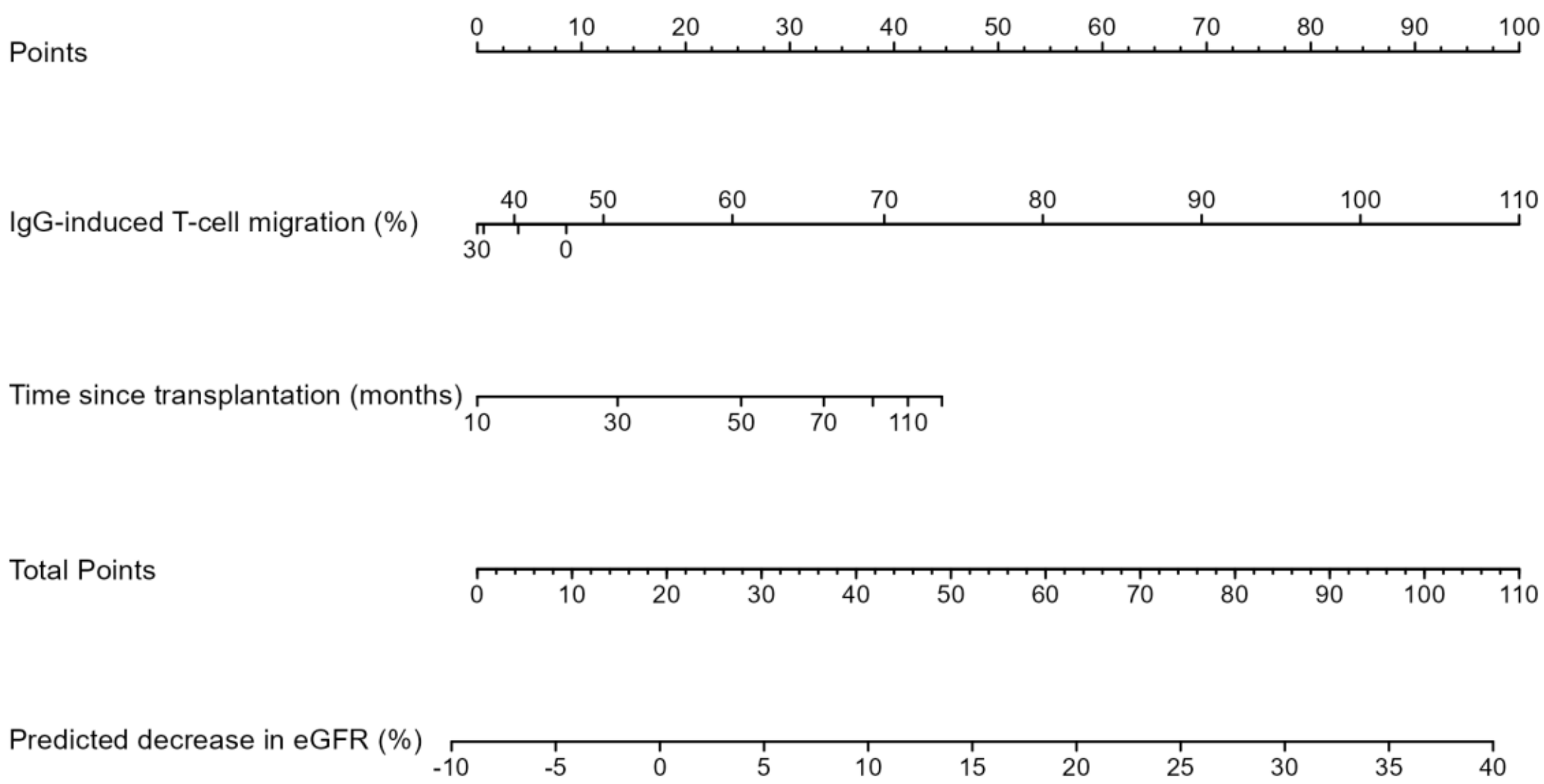

Supplementary Figure S4. Nomogram derived from the multivariable model shown in Figure 7. The nomogram is provided as a visual summary of the fitted model and illustrates the potential contribution of T-cell migration and time since transplantation to the estimated eGFR decline. The model was developed in 40 patients and requires external validation before potential clinical application. The migration axis is displayed on two lines to accommodate the non-monotonic shape of the fitted effect at low migration values.

Supplementary Table S1. Sensitivity analysis for the 600 U/mL exclusion. Re-fitting the final model with the excluded sample re-included gives n = 41 and an apparent R^2 of 0.463, against n = 40 and 0.467 for the model reported in the main text; the non-linear effect of T-cell migration remains significant in both ($p = 0.008$).

	Final model (outlier excluded; reported in the main text)	Sensitivity analysis (outlier re-included)
Patients contributing to the model, n	40	41
Apparent R^2	0.467	0.463
IgG-induced T-cell migration, overall p	0.0001	< 0.0001
IgG-induced T-cell migration, non-linear p	0.008	0.008
Time since transplantation, overall p	0.018	0.018

Both columns are ordinary least-squares models of the percentage decrease in eGFR on IgG-induced T-cell migration and time since transplantation, each entered as a restricted cubic spline with 3 knots. p-values are from the analysis of variance of the fitted model. The excluded sample had an anti-ETAR concentration of 663 U/mL, approximately twice the next highest value in the cohort.
